# Stimulation of Activin A/Nodal signaling is insufficient to induce definitive endoderm formation of cord blood-derived unrestricted somatic stem cells

**DOI:** 10.1186/scrt57

**Published:** 2011-04-04

**Authors:** Caitlin E Filby, Robert Williamson, Peter van Kooy, Alice Pébay, Mirella Dottori, Ngaire J Elwood, Faten Zaibak

**Affiliations:** 1 Early Development and Disease, Murdoch Childrens' Research Institute, Royal Children's Hospital, Flemington Rd, Parkville, VIC 3052, Australia; 2 Department of Paediatrics, University of Melbourne, Grattan Street, Parkville, VIC 3052, Australia; 3 Stem Cells and Cancer Division, Walter and Eliza Hall Institute of Medical Research, 1G Royal Parade, Parkville, VIC 3052, Australia; 4 Department of Pathology, University of Melbourne, Grattan Street, Parkville, VIC 3052, Australia; 5 Centre for Neuroscience and Department of Pharmacology, University of Melbourne, Grattan Street, Parkville, VIC 3052, Australia

## Abstract

**Introduction:**

Unrestricted somatic stem cells (USSC) derived from umbilical cord blood are an attractive alternative to human embryonic stem cells (hESC) for cellular therapy. USSC are capable of forming cells representative of all three germ line layers. The aim of this study was to determine the potential of USSC to form definitive endoderm following induction with Activin A, a protein known to specify definitive endoderm formation of hESC.

**Methods:**

USSC were cultured for (1) three days with or without 100 ng/ml Activin A in either serum-free, low-serum or serum-containing media, (2) three days with or without 100 ng/ml Activin A in combination with 10 ng/ml FGF4 in pre-induction medium, or (3) four days with or without small molecules Induce Definitive Endoderm (IDE1, 100 nM; IDE2, 200 nM) in serum-free media. Formation of definitive endoderm was assessed using RT-PCR for gene markers of endoderm (Sox17, FOXA2 and TTF1) and lung epithelium (surfactant protein C; SPC) and cystic fibrosis transmembrane conductance regulator; CFTR). The differentiation capacity of Activin A treated USSC was also assessed.

**Results:**

Activin A or IDE1/2 induced formation of Sox17+ definitive endoderm from hESC but not from USSC. Activin A treated USSC retained their capacity to form cells of the ectoderm (nerve), mesoderm (bone) and endoderm (lung). Activin A in combination with FGF4 did not induce formation of Sox17+ definitive endoderm from USSC. USSC express both Activin A receptor subunits at the mRNA and protein level, indicating that these cells are capable of binding Activin A.

**Conclusions:**

Stimulation of the Nodal signaling pathway with Activin A or IDE1/2 is insufficient to induce definitive endoderm formation from USSC, indicating that USSC differ in their stem cell potential from hESC.

## Introduction

Unrestricted somatic stem cells (USSC) are a population of stem cells that can be isolated from umbilical cord blood at birth. USSC have been shown to form the following cell types: nerve (ectoderm); heart, cartilage, bone, fat and blood (mesoderm); and liver and lung (endoderm) [1,2]. Although USSC have been shown to form cells representative of all three germ line layers [1,3,4], it is unclear whether USSC have an identical or a restricted capacity when compared to human embryonic stem cells (hESC).

Cord blood-derived USSC are an attractive source of cells for therapy, as unlike hESC, they can be obtained noninvasively at the time of birth, and have been shown not to form teratomas in animal models [1,5]. Specific factors or culture conditions cause USSC to be differentiated into progenitors of many cell types. Our research group is investigating the potential of USSC to form endodermal stem cells that may be useful for the treatment of cystic fibrosis and other diseases that affect the lung, liver, pancreas, thyroid and gut.

The developing endoderm gives rise to the lung, pancreas, liver, thyroid and gastrointestinal tract. Although the precise mechanisms are yet to be elucidated, it is apparent that the Nodal signaling pathway is involved in normal endoderm specification during embryonic development [6,7] as well as in the *in vitro *differentiation of stem cells into endoderm progenitors. Activin A is a protein known to stimulate the Nodal signaling pathway. Human ESCs [8-10] and induced pluripotent stem cells (iPSC) [11], as well as mouse ESCs [12], respond to high levels (100 ng/ml) of Activin A by commitment to Sox17+ definitive endoderm (DE). On the other hand, low levels of Activin A permits maintenance of pluripotency in stem cells [12,13]. In addition, stimulation of the Nodal signaling pathway in hESC using small molecules such as the Induce Definitive Endoderm molecules (IDE1 and IDE2) has also been shown to specify DE formation [14]. IDE1/2 induce phosphorylation of Smad2, a key component of the Nodal signaling pathway [14]. In the current study, we tested whether high levels of Activin A (alone or in combination with Fibroblast Growth Factor 4; FGF4), or IDE1/2 in a low serum or serum-free media causes USSC to form DE. We show that unlike ESCs, USSC do not respond to Activin A or IDE1/2 induction.

## Materials and methods

### Isolation and culture of USSC

Cord blood was collected with informed consent and USSC lines were successfully generated and the phenotype of the lines characterized as described previously, following a protocol approved by the University of Melbourne and the Royal Women's Hospital Human Ethics Review Committees (HREC No. 050886) [1,4,15]. The USSC lines used in this study were negative for CD34, CD24, CD31, CD45 and MHCII, and positive for CD44, CD71 and MHCI. The multilineage capacity of the lines was confirmed by differentiation into cells representative of the three germ line layers.

Cryopreserved USSC were thawed rapidly at 37°C and added to stem cell proliferation medium (SCPM), consisting of low-glucose Dulbecco's modified Eagle Medium (DMEM: Lonza, Walkersville, MD, USA) supplemented with 30% fetal calf serum (FCS; Hyclone, Logan, UT, USA), 0.1 mg/ml penicillin, 100 U/ml streptomycin (Invitrogen, Chadsworth, CA, USA) and 2 mM ultra glutamine (Lonza). Cells were centrifuged (five minutes, 1,500 rpm), the supernatant discarded and the cell pellet resuspended in 10 ml of SCPM; this was repeated once. Resuspended cells (approximately 10^6^) were transferred to 75 cm^2 ^flasks and cultured at 37°C in 5% CO_2_. The medium was changed the day after thawing, and then every four to five days. Cells were passaged once they reached 80% confluence; medium was removed, cells were washed with phosphate-buffered saline (PBS), and then dissociated by incubation with 4 ml of 0.25% v/v trypsin/0.02% v/v EDTA at 37°C for 5 to 10 minutes. Trypsin was neutralised by the addition of 2 ml 4% human serum albumin (Albumex^® ^20, CSL Limited, Parkville, VIC, Australia) in PBS. Cell numbers were determined using the Countess (Invitrogen), by analysing 10 μl of a 50% cell suspension in trypan blue. Dissociated cells were centrifuged (five minutes, 1,500 rpm), the supernatant discarded and the pellet resuspended in SCPM to obtain 10^6 ^cells/10 ml which were plated in 75 cm^2 ^flasks (unless otherwise indicated). USSC used in this study were seeded for testing at passage five to passage nine.

### Determination of USSC survival in low-serum media

To avoid problems due to variation in biologicals such as fetal calf serum (FCS) or Matrigel, Activin A induction was performed in low serum or serum-free SCPM. To determine if USSC survive these culture conditions, three USSC cell lines were seeded in duplicate in SCPM containing varying concentrations of FCS: 0% (serum-free SCPM), 1%, 2% and 30%. Cells (0.4 × 10^6 ^total, 4.1 × 10^4 ^cells/cm^2^) were seeded near confluency in six-well plates in 2.5 ml of media/well. Cells were observed and after seven days were dissociated, counted and cell pellets snap frozen on dry ice for later determination of Activin A receptor mRNA levels. Cell counts were calculated and expressed as a percentage of the number of cells originally seeded.

### Activin A induction of USSC

Lyophilized carrier-free recombinant human Activin A (Catalogue No. 338-AC/CF, R&D Systems Inc., Minneapolis, MN, USA) was reconstituted in sterile PBS to a stock concentration of 100 μg/ml. To ensure consistency in induction, all vials of Activin A that were used were from one of two batches. The same batch was used across experiments that were directly compared to one another. At least three USSC lines and two hESC lines consisting of five colonies were tested for their response to Activin A.

USSC at passage five were seeded into one of three different treatment groups: SCPM containing 30% FCS (*Normal serum control*); SCPM containing 0 (Day 1), 0.2% (Day 2) and 2% (Day 3) FCS (*Low serum control*); SCPM containing 100 ng/ml Activin A and 0% (Day 1), 0.2% (Day 2) and 2% (Day 3) FCS (*Activin A*) (Additional File 1) as previously described [8].

Cells were initially seeded in the appropriate media (either SCPM containing 30% or 0% FCS), depending on the treatment group to which each was assigned. The number of cells seeded depended on the treatment group, as culturing USSC in serum-free SCPM results in a 50% loss in cell number. Hence, cells were seeded in 145 cm^2 ^dishes containing 25 ml media at a density of 3.3 × 10^4 ^cells/cm^2 ^when cultured in serum-free SCPM or 2.0 × 10^4 ^cells/cm^2 ^when cultured in standard SCPM. After 24 hr, the seeding media was replaced with the appropriate treatment media and cells were incubated for 72 hr.

Following the induction period, USSC were dissociated, counted and a proportion of the cells (5 × 10^5 ^cells per treatment) were centrifuged and the cell pellets frozen for RNA extraction. The remaining cells from each treatment group were seeded in triplicate. Each culture was divided into two, one of which was tested using each of the three differentiation assays (described below) to allow undifferentiated and differentiated samples of each triplicate to be compared. For RNA extraction, cells were seeded at 5 × 10^5 ^cells in six-well plates. For immunohistochemistry, cells were seeded at 4.2 × 10^4 ^cells in chamberslides. For Alizarin Red staining, cells were seeded at 2 × 10^4 ^cells in 12-well plates.

Several other variations of this Activin A induction strategy were attempted, including culturing the USSC for the entire induction period in either serum-free SCPM or SCPM containing 2% FCS supplemented with 100 ng/ml Activin A.

### USSC differentiation assays

The following differentiation assays were performed on Activin A treated USSC to determine whether Activin A treatment affected USSC multipotency. To induce mesoderm differentiation, cells were cultured for 10 days in proliferation medium containing DAG (10^-7 ^M dexamethasone, 50 μg/ml ascorbic acid and 10 mM glycerol-2-phosphate disodium salt (all from Sigma-Aldrich Castle Hill, NSW, Australia) as described by Jaiswal *et al. *[16]). Differentiated cells were pelleted for RNA extraction and PCR analysis (details below) or were stained with Alizarin Red to detect calcium deposits from bone cells, indicating successful mineralization. For Alizarin Red staining, cells were fixed in 70% ice-cold ethanol (4°C, 10 minutes) and stained with 1% Alizarin Red S (Sigma-Aldrich) in distilled water, pH 4.2 (room temperature, 10 minutes). Cells were washed in distilled water, layered with PBS (calcium and magnesium free) and images were captured using a using Leica DMIRB inverted microscope and AxioVision 4.2 software (Carl Zeiss AG, Oberkochen, Germany).

To demonstrate neuronal differentiation capacity, USSC were cultured with a modified differentiation protocol for 12 days [15]. Briefly, cells were seeded in fibronectin-coated wells (10 μg/ml poly-D-lysine, Sigma-Aldrich; 10 μg/ml fibronectin, Becton Dickinson, Franklin Lakes, NJ, USA) and cultured in neural basal medium (NBM) consisting of Neurobasal A with 2% B-27, 1% insulin-transferrin-selenium-A, 1% N2, 2 mM L-glutamine, 100 U/ml penicillin and 0.1 mg/ml streptomycin (all sourced from Invitrogen), supplemented with basic fibroblast growth factor epidermal growth factor (both 20 ng/ml, R&D Systems). Media were changed every two days. At the end of the differentiation protocol, cells were collected for PCR analysis. Successful differentiation was determined by RT-PCR for expression of Nestin and Pax6.

To test for epithelial differentiation, cells were grown in small airway growth medium (SAGM; Lonza) as described by Berger *et al. *[17]. USSC were seeded at 5 × 10^5 ^cells per well in a six-well plate and then cultured in SAGM for eight days. Medium was changed every two days. Successful differentiation was determined by RT-PCR for expression of surfactant protein C (SPC).

### Combined Activin A and FGF4 induction of USSC

USSC (USSC1 at passage 6, USSC2 at passage 9) were seeded at 5 × 10^3 ^cells/cm^2 ^in six-well plates in 3.5 ml of SCPM and were cultured for 72 hr to reach 80% confluency. For both USSC lines, the following treatments were performed in triplicate: SCPM containing 30% FCS (*SCPM control*); Pre-induction media (PIM; DMEM low glucose:Hams F12 Salts containing Glutamax, Penicillin/streptomycin, 15 mM Hepes Buffer, 1 mM Sodium Pyruvate, 1 g/L BSA, 5 mM nicotinamide,1.5 μg/ml ethanolamine as described in Waclawczyk *et al. *[18]) containing 0% (Day 1), 0.5% (Days 2 to 3) FCS (*PIM control*); PIM containing 100 ng/ml Activin A and 0% FCS (Day 1), with the addition of 10 ng/ml FGF4 (Catalogue No. 235-F4/CF, R&D Systems Inc.) and 0.5% (Days 2 to 3) (*Activin A + FGF4*) as previously described [18].

Following the induction period, USSC were dissociated, centrifuged and cell pellets frozen for RNA extraction.

### Controls for Activin A or FGF4 induction

To confirm Activin A activity, hESC (Envy or H9; approved by the University of Melbourne Human Research Ethics Committee, HREC No. 0605017.5) were used as a positive control for Sox17 induction. hESC (five colonies per replicate; two replicates) were cultured in Roswell Park Memorial Institute medium (RPMI) containing 100 ng/ml Activin A for 72 hr as described previously [8].

To confirm FGF4 activity, NIH 3T3 fibroblasts were used as a positive control; FGF4 has a mitogenic effect on fibroblasts [19]. NIH 3T3 fibroblasts were seeded at 3 × 10^3 ^cells/cm^2 ^in six-well plates in DMEM high glucose (Catalogue No. 12100046, Invitrogen) containing 10% heat-inactivated serum and 3.4 g/L HCO_3_. After 24 hr, the media was replaced with fresh media with/without FGF4 (0, 5, 10, 20 or 40 ng/ml; in triplicate). Following 48 hr of FGF4 induction, cells were dissociated and enumerated to determine the effect of FGF4 on total cell numbers.

### IDE1 or IDE2 induction of USSC and hESC

A small sample of IDE1 and IDE2 (10 nM in DMSO) were kindly supplied by Professor Stuart Schreiber. USSC were seeded at high density (10^5 ^cells/cm^2^) in SCPM in 48-well plates to ensure cell numbers high enough to detect mRNA levels and to minimize media volumes given the limiting amount of IDE1/2. Colonies (one to two) of hESC (Envy, passage 94) were seeded into wells of an eight-well chamberslide, on a feeder layer of mouse embryonic fibroblasts, as previously described [20]. Induction of USSC and hESC occurred simultaneously with the same batch of IDE1/2. Two replicates were assigned to each treatment group for each cell type, except cells treated with IDE2, as insufficient sample permitted only one replicate per cell type. USSC were treated for four days with (1) Normal USSC media (SCPM), (2) Control media (RPMI) (3) RPMI containing 100 nM IDE1, or (4) RPMI containing 200 nM IDE2. hESC were treated with (1) Normal hESC media with serum, as previously described [20], (2) Control media (RPMI) (3) RPMI containing 100 nM IDE1, or (4) RPMI containing 200 nM IDE2.

Cell morphology was observed and representative photographs taken. After four days, USSC and hESC were dissociated and the cell pellets frozen for RNA extraction. Immunohistochemistry and differentiation assays were not performed as the small sample of IDE1/2 necessitated that only very small numbers of cells could be treated.

### RNA Extraction and PCR

Total RNA was extracted using the RNeasy Plus Mini Kit (Activin A and low serum study; Qiagen, Germantown, MD, USA) or RNeasy Plus Micro Kit (IDE study; Qiagen) according the manufacturer's protocol. RNA was quantified using a NanoDrop 1000 Spectrophotometer. cDNA was synthesized from 250 ng RNA using random hexamers according to the manufacturer's protocol (Superscript III kit, Invitrogen). Real-time PCR reactions (25 μl) contained ABI 2 × SYBR Green PCR Master Mix (Applied Biosystems, Mulgrave, VIC, Australia, containing SYBR Green I Dye, AmpliTaq Gold DNA Polymerase, dNTPS with dUTP, passive reference and optimized buffer components), 0.4 μM primers and cDNA template. Amplification conditions for real-time PCR reactions were: one cycle at 95°C for 10 minutes; 40 cycles at 95°C for 30 sec, 60°C for 30 sec and 72°C for 1 minute; followed by melt curve analysis (50 to 90°C, rising 1°C per 5 sec). Standard and nested PCR reactions (25 μl) contained 10 × PCR buffer, 5 × Q solution, 200 nM dNTPs, 400 nM of each primer and 0.625 U Taq polymerase (Taq Core Kit, Qiagen, Germantown, MD, USA). Amplification conditions for standard and nested PCR reactions were: one cycle at 94°C for 2 to 3 minutes; 30 (nested) to 35 (standard) cycles at 94°C for 30 sec, 54 to 60°C for 30 sec and 72°C for 1 minute; followed by extension at 72°C for 2 to 10 minutes. Primer sequences and annealing temperatures are detailed in Table 1. Positive controls were either spontaneously differentiated hESC or human bronchial epithelial cells. Gene expression was analyzed for the following genes: Sox17, FOXA2, TTF1, surfactant protein C (SPC), cystic fibrosis transmembrane conductance regulator (CFTR) (endoderm/lung markers), Nestin, Pax6 (ectoderm markers), Mixl1 and Brachyury (mesoderm markers).

**Table 1:** Primers used for PCR in this study

Gene	Primer sequences 5'-3'		PCR type	Annealing temp. (°C)	Cycle number	Amplicon size (bp)
***ACVR1a***	ACVR1a-F	TAA GCG TCA CAC TGC CAA AG	Real-time	60	40	89
	ACVR1a-R	GTC ACT GGG GTA CTC GGA GA				
***ACVR1b***	ACVR1b-F	TGC AAC AGG ATC GAC TTG AG	Real-time	60	40	121
	ACVR1b-R	TGA GGA CAG GAG GAA CAC C				
***ACVR1c***	ACVR1c-F	TGG TCT GGC ACA CCT TCA TA	Real-time	60	40	137
	ACVR1c-R	TCA CAG CCA ACC CTA AGT CC				
***ACVR2a***	ACVR2a-F	GCG TTT GCC GTC TTT CTT AT	Real-time	60	40	138
	ACVR2a-R	TGT CAC CAT AAC ACG GTT CAA				
***ACVR2b***	ACVR2b-F	CTC CTC TGG GGA TCG CTG T	Real-time	60	40	137
	ACVR2b-R	CTT GTC CTG CTC GCC TTC				
***Brachyury***	Brachyury-F	GAA CCA GCA CCC TGT GTC CAC	Standard	55	38	608
	Brachyury-R	GCC ACG ACA AAA AGT CAC TGC				
***CFTR***	CFTR-F	ACT TTA AAG CTG TCA AGC CGT G	Standard	35	30	1051
	CFTR-R	CAT CAT AGG AAA CAC CAA A				
***CFTR (nested)***	CFTR-NF	AGC TGT CAA GCC GTG TTC TAG ATA	Nested	45	30	140
	CFTR-NR	ATG AGG AGT GCC ACT TGC AAA				
***FOXA2***	FOXA2-F	GGG AGC GGT GAA GAT GGA	Standard	55	35	89
	FOXA2-R	TCA TGT TGC TCA CGG AGG AGT A				
***GAPDH***	GAPDH-F	ATG GAG AAG GCT GGG GCT C	Real-time and standard	60	40	196
	GAPDH-R	AAG TTG TCA TGG ATG ACC TTG				
***MixL1***	MixL1-F	CCG AGT CCA GGA TCC AGG TA	Standard	55	35	58
	MixL1-R	CTC TGA CGC CGA GAC TTG G				
***Nestin***	Nestin-F	CAG CTG GCG CAC CTC AAG ATG	Standard	55	35	209
	Nestin-R	AGG GAA GTT GGG CTC AGG ACT GG				
***SPC***	SPC-F	AGC CAG AAA CAC ACG GAG AT	Standard	57	30	456
	SPC-R	AGT GGA GCC GAT GGA GAA G				
***SPC (nested)***	SPC-NF	AAC GCC TTC TTA TCG TGG TG	Nested	57	30	313
	SPC-NR	GTG AGA GCC TCA AGA CTG G				
***Sox17***	Sox17-F	GGC GCA GCA GAA TCC AGA	Real-time	60	40	61
	Sox17-R	CCA CGA CTT GCC CAG CAT				
***TTF1***	TTF1-F	ACC AGG ACA CCA TGA GGA AC	Standard	50	38	116
	TTF1-R	GCT CAT GTT CAT GCC GCT				

Efficiency correlations confirmed that the efficiency of Sox17 was similar to that of GAPDH. The ΔΔCT method was then used to determine the gene expression levels, corrected for GAPDH.

### ACVR Immunohistochemistry

USSC1 and USSC2 were seeded in duplicate in eight-well chamberslides (Permanox slides, Cat # 177445, Labtek, Brendale, QLD, Australia) in SCPM containing 30% FCS (2.0 × 10^4 ^cells/cm^2^) or in SCPM containing 0% FCS (3.3 × 10^4 ^cells/cm^2^). Cells were fixed by removing the media, performing two times five-minute washes in PBS, then incubating the cells in 4% paraformaldehyde/PBS at 4°C for five minutes. The paraformaldehyde was removed and cells washed in PBS and stored at 4°C in PBS+ until commencing immunohistochemistry.

Cells were permeabilised in 0.1% Triton-x in tris-buffered saline (TBS) for 10 minutes, washed in dH_2_O before endogenous peroxidases were quenched in 3% H_2_O_2 _for 5 minutes. Cells were washed in dH_2_O and TBS before the cells were blocked with 0.5% BSA/TBS for 30 minutes. Cells were then incubated with primary antibody (all goat polyclonal IgG; 1:50; ACVR1b Cat. # sc-5665; ACVR2a Cat. # sc-5667; ACVR2b Cat. # sc-11984, Santa Cruz Biotechnology, Santa Cruz, CA, USA) or rabbit IgG (Cat. # sc-2027; 1:1000, Santa Cruz Biotechnology, Santa Cruz, CA, USA) in 0.5% BSA/TBS at 4°C overnight.

Cells were washed in TBS before incubation with the secondary antibody (DAKO polyclonal rabbit anti-goat Ig Biotinylated Cat. # E0466) for 1 hr. Cells were washed in TBS before being incubated with Avidin-Biotinylated enzyme Complex (Vectastain, Vector Laboratories, Burlingame, CA, USA) for 30 minutes. Cells were washed in TBS and 3,3'-diaminobenzidine (DAB) containing 10 μl/ml 6% H_2_O_2 _was added to the sections to induce formation of a specific signal. Cells were washed in dH2O, stained with Harris' Haematoxylin and mounted (Invitrogen Prolong Gold Aqueous mountant). TCam2 seminoma cells were used as a positive control.

### Statistical analyses

The following differences were determined by a two-way unpaired t-test (assuming equal variances): 1) differences in cell proliferation indices between USSC cultured in 0%, 1%, 2% or 30% FCS and, 2) differences in Sox17 or Brachyury mRNA levels between untreated, Activin A treated and spontaneously differentiated hESC and normal serum, low serum and Activin A treated USSC. Results were considered statistically significant at *P *< 0.05.

## Results

### Determination of USSC survival in serum-free culture media

Prior to inducing USSC with Activin A, the survival potential of USSC in a low serum or serum-free environment was assessed. USSC seeded at confluence (4.2 × 10^4 ^cells/cm^2^) and grown in SCPM containing 0%, 1% and 2% retained 51.2%, 63.0% and 87.8% of the initial seeding density of cells after seven days while cells grown in 30% FCS increased (149.5%) (Additional File 2), indicating that USSC can survive in low serum or serum-free environments for a short period of time. When seeded at 1.3 × 10^4 ^cells/cm^2 ^the number of viable USSC is normally approximately 300% of initial seeding density after five days of growth in SCPM containing 30% FCS. In the current experiment, USSC were seeded in media containing 30% FCS at confluence; therefore, only a slight increase in the initial seeding density was obtained.

### Activin A treatment of USSC does not induce DE formation

**Figure 1:**
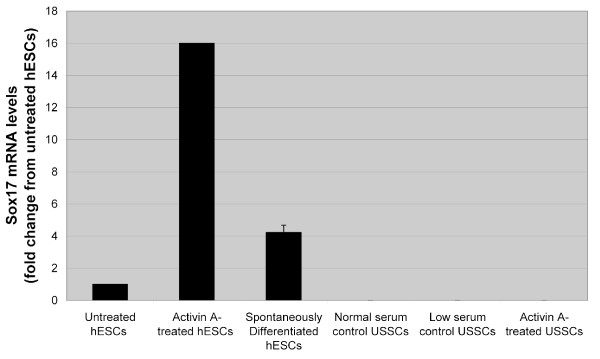
**Sox17 mRNA levels following Activin A induction**. Sox17 mRNA levels for untreated and Activin A treated USSC lines and untreated, spontaneously differentiated and Activin A treated hESC. Sox17 mRNA levels were very low in untreated and Activin A treated USSC lines (0.001-fold of untreated hESC). Sox17 mRNA levels were low in untreated hESC, moderately low in spontaneously differentiated hESC and significantly up-regulated in Activin A treated hESC.

The three USSC lines tested did not express mRNA for the DE marker Sox17 either before or after treatment with 100 ng/ml Activin A using a step-wise increase in FCS over three days (Figure 1), indicating that Activin A treatment of USSC does not induce the production of Sox17+ DE. On the other hand, Sox17 mRNA levels are very low in undifferentiated Envy hESC, increase 4-fold in spontaneously differentiated Envy hESC and are up-regulated 16-fold in Envy hESC treated with the same Activin A induction strategy. Activin A treatment of USSC using media containing either 0% or 2% FCS was also assessed; however, neither of these strategies resulted in up-regulation of Sox17 expression (data not shown).

### USSC retain the capacity for multipotency following Activin A induction

**Figure 2:**
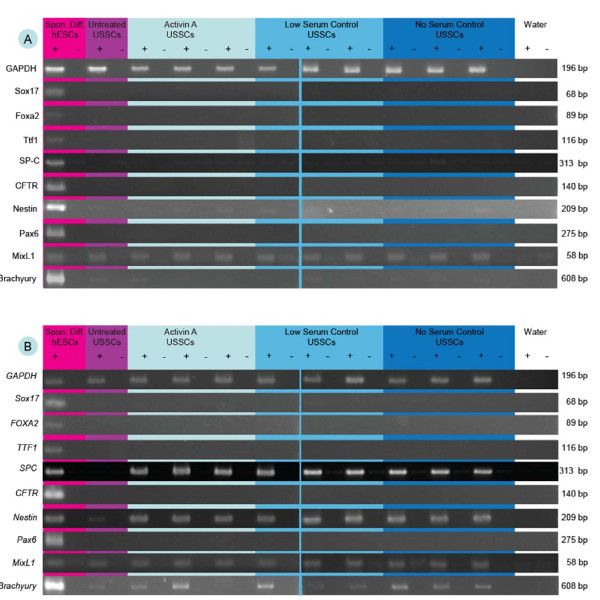
**Markers of endoderm, ectoderm and mesoderm differentiation in Activin A treated USSC following directed differentiation**. Representative gels depicting mRNA levels of the housekeeping gene GAPDH, endoderm markers Sox17, FOXA2, TTF-1, SPC and CFTR, ectoderm markers Nestin and Pax6 and mesoderm markers MixL1 and Brachyury. mRNA levels were determined in Activin-A treated USSC, low serum control USSC and serum-free controls before (Figure 2a) and following (Figure 2b) differentiation into cells representative of each of the three germline layers. Spontaneously differentiated hESC and human bronchial epithelial cells were used as positive controls and undifferentiated USSC were used as negative controls. + depicts RT positive cDNA, - depicts RT negative controls.

**Figure 3:**
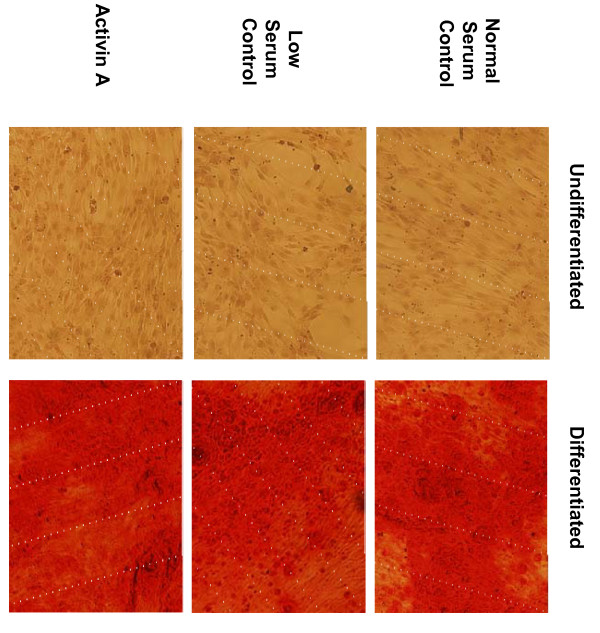
**Alizarin red staining of USSC**. Representative light micrographs depicting calcium deposits detected by Alizarin red staining in *Normal serum control*, *Low serum control *and *Activin A *treated USSC (100×) before and after mesoderm differentiation.

The mRNA levels of markers of endoderm, ectoderm and mesoderm were not different between untreated and Activin A-treated USSC either before or following directed differentiation (Figure 2), indicating that USSC retain their capacity for multipotency following Activin A induction. Specifically, mRNA for Sox17, FOXA2, TTF-1 and CFTR were not detected in untreated or Activin A treated USSC either prior to or following endodermal differentiation with SAGM. The level of SPC mRNA was similar in both untreated and Activin A treated USSC following endodermal differentiation with SAGM. mRNA for Nestin was detected in both untreated and Activin A treated USSC, and was upregulated after ectodermal differentiation. On the other hand, Pax6 mRNA was not detected in USSC regardless of Activin A treatment or ectodermal differentiation. Similarly, MixL1 mRNA was detected in USSC regardless of Activin A treatment or mesodermal differentiation, while Brachyury was only detected in some of the USSC lines studied and only following mesodermal differentiation. USSC retained the capacity to form cells capable of mineralization, as determined by the detection of calcium deposits using Alizarin red staining (Figure 3).

### Combined treatment of USSC with Activin A and FGF4 does not induce DE formation

**Figure 4:**
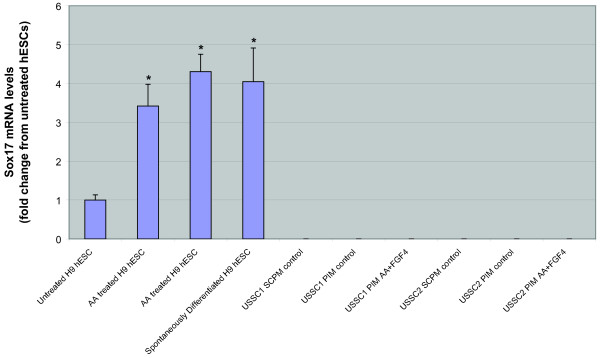
**Sox17 mRNA levels following combined Activin A and FGF4 induction**. Sox17 mRNA levels for untreated and Activin A and Fibroblast Factor 4 treated USSC lines and untreated, spontaneously differentiated and Activin A treated hESC. Sox17 mRNA levels were very low in untreated USSC cultured in SCPM or PIM and in USSC lines treated with Activin A and Fibroblast Factor 4. Sox17 mRNA levels were low in untreated hESC, and significantly up-regulated in Activin A treated hESC and in spontaneously differentiated hESC.

**Figure 5:**
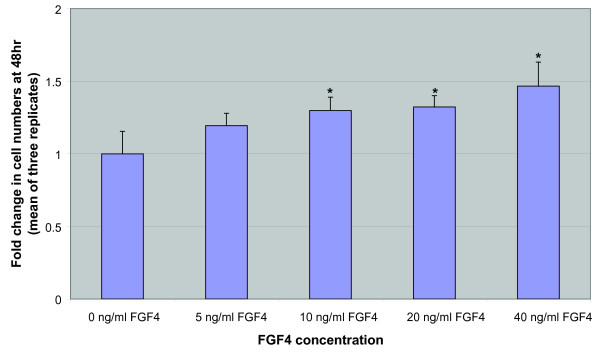
**Fibroblast Growth Factor 4 is active**. Fold change in NIH 3T3 cell numbers following treatment with Fibroblast Growth Factor 4. A dose-dependent increase in NIH 3T3 cell numbers was observed with an increase in Fibroblast Growth Factor 4 concentration, indicating that the growth factor is active.

The two USSC lines tested did not express Sox17 before or after treatment with 100 ng/ml Activin A and 10 ng/ml FGF4 indicating that Activin A in combination with FGF4 does not induce the production of Sox17+ DE from USSC (Figure 4). Activin A was shown to induce Sox17 expression in H9 hESC, indicating that the Activin A was active. Similarly, FGF4 was shown to have a mitogenic effect on NIH 3T3 cells, resulting in a dose-dependent increase in cell numbers following 48 hr of FGF4 treatment (Figure 5), indicating that the FGF4 was active.

### IDE1/2 treatment of USSC does not induce DE formation

**Figure 6:**
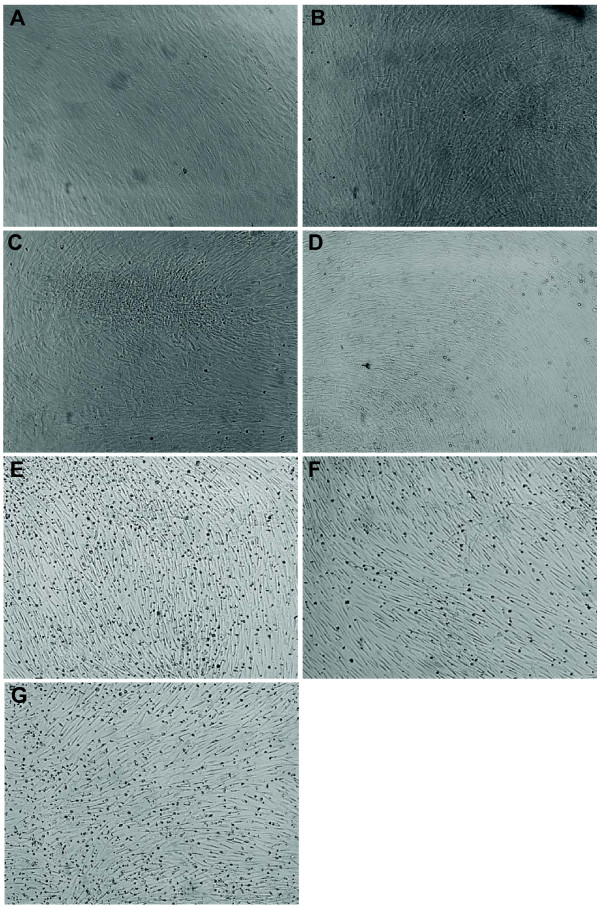
**Representative micrographs of IDE1/2-treated USSC**. USSC grown in SCPM (**A, B**), RPMI (**C, D**), or RPMI containing 100 nM IDE1 (**E, F**) or 200 nM IDE2 (**G**) (100×). A proportion of USSC treated with IDE1/2 developed a rounded morphology (E, F, G).

**Figure 7:**
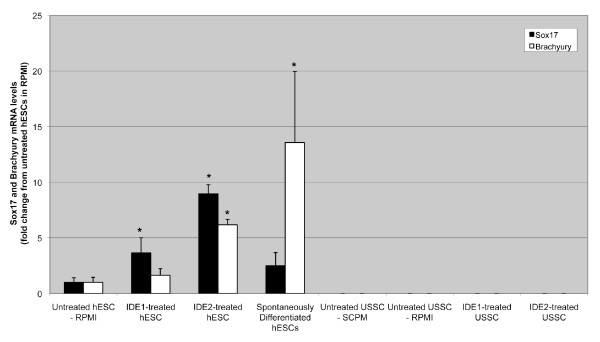
**{Sox17 and Brachyury levels following IDE1/2 induction**. Sox17 (black bars) and Brachyury (white bars) mRNA levels for untreated and IDE1/2 treated USSC lines and untreated, spontaneously differentiated and IDE1/2 treated hESC. Sox17 and Brachyury mRNA levels were very low in untreated and IDE1/2 treated USSC lines. Sox17 and Brachyury mRNA levels were low in untreated hESC. Sox17 mRNA was significantly up-regulated in IDE1/2 treated hESC. Brachyury expression was significantly up-regulated in IDE2 treated hESC and spontaneously differentiated hESC. Data represent the mean ± SD of three PCR replicates from two cell culture replicates for each experimental treatment (*P < 0.05).

Following induction with IDE1/2, a proportion of USSC developed a more rounded morphology (Figure 6). Neither Sox17 nor Brachyury could be detected in untreated or IDE1- or IDE2-treated USSC (Figure 7), indicating that IDE1/2 do not induce DE or mesendoderm formation of USSC. On the other hand, the level of Sox17 mRNA in Envy hESC is moderate in undifferentiated Envy hESC, elevated in spontaneously differentiated Envy hESC, and significantly increased following treatment with IDE1 or IDE2. Brachyury was also moderately expressed in Envy hESC and highly expressed in spontaneously differentiated Envy hESC. IDE2 but not IDE1 up-regulated the level of Brachyury mRNA in Envy hESC.

### USSC express Activin A receptors (ACVR)

**Figure 8:**
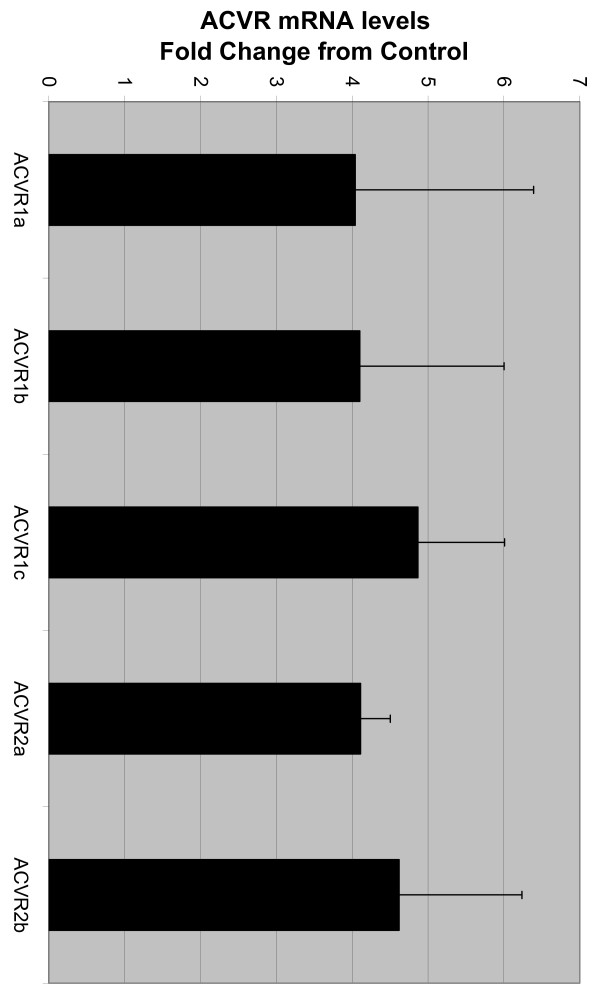
**Activin receptor (ACVR) mRNA levels**. ACVR mRNA levels for USSC cultured in SCPM containing 0% FCS, expressed as fold change from mRNA levels in USSC cultured in control (SCPM containing 30% FCS). All ACVR mRNA levels are significantly increased in USSC cultured in 0% FCS, compared to USSC cultured in SCPM containing 30% FCS (*P < 0.05). Data represent the mean ± SD of three PCR replicates from four cell lines for each experimental treatment.

**Figure 9:**
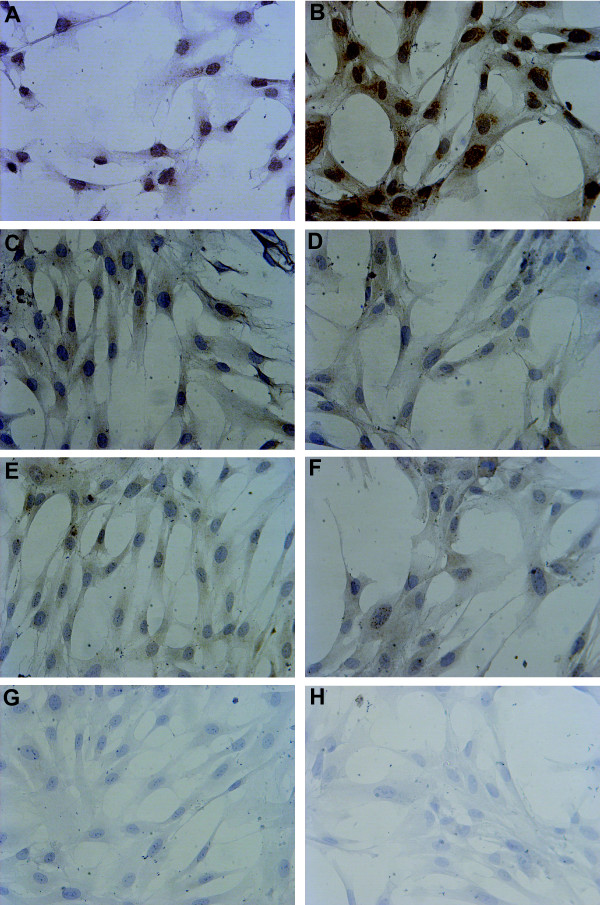
**Activin receptor (ACVR) protein levels**. Representative light micrographs (400×) depicting the localization of ACVR (brown staining) in USSC grown in SCPM. Nuclei are counterstained blue with Haematoxylin. USSC 1 (left column) and USSC 2 (right column) expressed ACVR1b (**A, B**), ACVR2a (**C, D**) and ACVR2b (**E, F**). No staining was detected in the IgG controls (**G, H**).

USSC express mRNA for ACVR1a, ACVR1b, ACVR1c, ACVR2a and ACVR2b when cultured in SCPM containing 30% FCS as determined by RT-PCR. When cultured in SCPM containing 0% FCS, the mRNA levels for ACVR1a, ACVR1b, ACVR1c, ACVR2a and ACVR2b increases approximately four-fold (Figure 8; *P *< 0.05). Immunohistochemistry analysis revealed that USSC also express ACVR1b, ACVR2a and ACV2b protein when cultured in SCPM containing 30% FCS (Figure 9). USSC continue to express ACVR1b, ACVR2a and ACVR2b protein when cultured in serum free conditions (SCPM containing 0% FCS; Additional File 3).

## Discussion

The results of this study demonstrate that Activin A/Nodal signaling alone is insufficient to induce formation of Sox17+ endoderm from USSC. Activin A induction (100 ng/ml, 72 hrs) was tested in three independent experiments in triplicate, using media that was serum-free, contained low serum (2%) or which had an incremental increase in serum (0, 0.2 and 2%). Activin A treated USSC showed no change in their capacity to form nerve-like (ectoderm), bone-like (mesoderm) or lung-like (endoderm) cells, indicating that Activin A treatment alone does not direct differentiation of USSC. Small molecule induction of the Nodal signaling pathway using IDE1/2, which are highly efficient in inducing DE formation of hESC [14], also does not result in DE formation of USSC. Furthermore, we report that induction with Activin A in combination with FGF4 does not induce Sox17+ DE formation of USSC. Finally, we show that USSC express the ACVR and are therefore capable of responding to Activin A signals.

Unlike USSC, Activin A induction of hESC line Envy with an incremental increase in serum was sufficient to cause a 16-fold increase in Sox17 mRNA levels, a level of up-regulation that is similar to previous reports of Activin A-induced hESC endoderm formation [8]. Similarly, the H9 hESC line also showed increased Sox17 mRNA levels in response to Activin A induction. H9 showed a similar trend to Envy, although the magnitude was somewhat smaller. Hence, although there are differences between hESC lines, well characterized hESC lines such as Envy and H9 respond to growth factor stimulation in a relatively robust, repeatable manner with a similar trend in terms of expression of key markers of differentiation. Similarly, IDE1/2 induction resulted in up-regulation of Sox17 in hESC, confirming earlier reports that IDE1/2 induce formation of DE from hESC [14]. Although Brachyury mRNA was detected in untreated hESC, there was no discernible increase in Brachyury expression following IDE1/2 induction. This may indicate that if hESC pass through a mesendoderm intermediate that they may have already done so after four days of IDE1/2 induction.

Unlike hESC, USSC appear to be a heterogeneous population [21] (and our own observations), one that still requires further characterization. Indeed, here we report that USSC1 and USSC2 do not form Sox17+ cells when treated with Activin A in combination with FGF4. We report that the growth factors Activin A and FGF4 used in this study are active (as indicated by their effect on hESC and NIH 3T3 cells respectively) but do not induce Sox17 expression in USSC1 (derived in our laboratory) and USSC2 (derived in Gesine Kogler's laboratory). Our results disagree with those of Waclawczyk and Kogler *et al. *[18] who found that the same induction strategy induced formation of hepatocytes; those USSC expressed Sox17 mRNA at Day 3 of directed differentiation and cytoplasmic Sox17 protein at Day 5. The significance of the cytoplasmic localization of the transcription factor Sox17 in that study is unclear; it may indicate that although Sox17 was present it was not active. The results of the current study indicate that the combination of Activin A and FGF4 is not a robust induction protocol for inducing Sox17 expression in USSC.

There are a number of possibilities why USSC do not respond to Activin A signaling alone. Indeed, other authors have shown that Activin A-induced DE formation in hESC (1) can be enhanced by the addition of Wnt3a [10], sodium butyrate [22] or insulin transferrin-selenium (ITS) [9], or (2) can be achieved only by ensuring phosphoinositide 3-kinases (PI3K) are suppressed [23]. This study did not test these modified Activin A induction strategies, so none of these possibilities can be excluded. However, numerous studies (including the current study) show that Activin A alone is sufficient for endoderm formation from ESCs [8,12,24,25]. This study can only conclude that unlike in hESC, in USSC Activin A/Nodal signaling alone is insufficient to induce DE formation.

We show that USSC express the necessary receptors to respond to Activin A signaling. All USSC lines expressed all five ACVRs at the mRNA level and express ACVR1b, ACVR2a and ACVR2b at the protein level; the combination of these receptors is sufficient for functional Activin A signaling [26]. USSC may lack other functional components of the signaling cascade; further studies that may elucidate this issue include those that determine 1) receptor functionality using an Activin A fluorokine assay, 2) nuclear localization of Smad2 or 3) the baseline of Activin A signaling by addition of Activin A inhibitors such as Follistatin or Inhibin in USSC. Alternatively, interrogation of this signaling pathway may in fact reveal that USSC have all the required Activin A signaling components, but may have passed the developmental window in which they will respond to Activin A by forming DE.

Although Activin A induction in combination with other growth factors may be sufficient for specifying committed cell types such as hepatocytes [18], growth factors alone are unlikely to be sufficient for USSC-Sox17+ DE formation. We speculate that this may reflect that USSC, although capable of forming cells representative of all three germline layers, have a restricted differentiation potential compared to hESC. Indeed, USSC do not express Oct4 [4] but do express Klf4 and c-Myc [27], while hESC express all of the pluripotency markers. Furthermore Kluth *et al. *[21] propose that USSC may be derived from the fetal liver, hence USSC may have a greater propensity to form hepatocytes and may require stronger signals to revert back to less committed cell types such as DE progenitors. Hence, USSC may have a more restricted pluripotent capacity than initially thought and may require partial reprogramming to form endodermal progenitors.

## Conclusions

In conclusion, although cord blood-derived USSC have the potential to form cells derived from the endoderm, this study demonstrates that they do not form Sox17+ DE when treated with Activin A or IDE1/2; conditions that specify hESC endoderm differentiation. This indicates that USSC respond differently to hESC when induced with Activin A and that there is a fundamental difference in the multipotent capacity of these two types of stem cells. Clearly, further characterization of USSC is required in order to fully understand and realize the potential of USSC, and allow us to understand their position in the hierarchy of lineage of human stem cells.

## Abbreviations

ACVR: Activin A receptor; CFTR: cystic fibrosis transmembrane conductance regulator; DAB: 3,3'-diaminobenzidine; DE: definitive endoderm; FCS: fetal calf serum; FGF4: fibroblast growth factor 4; hESC: human embryonic stem cell(s); IDE: induce definitive endoderm; iPSC: induced pluripotent stem cell(s); ITS: insulin transferrin-selenium; PBS: phosphate-buffered saline; PIM: pre-induction media; SCPM: stem cell proliferation medium; USSC: unrestricted somatic stem cell(s)

## Competing interests

The authors declare that they have no competing interests.

## Authors' contributions

CF designed parts of the study, carried out the study, and wrote and edited the manuscript. PVK carried out initial experiments. RW and NE edited the manuscript and provided intellectual input into the study. AP and MD performed the ESC experiments. FZ devised the initial study, performed initial experiments and provided editorial input into the manuscript.

## Supplementary Material

Additional file 1 **Activin A induction strategy**. Cells were divided into one of three treatment groups; *Normal serum control*, *Low serum control *and *Activin A*. *Normal serum control *cells were seeded in SCPM containing 30% FCS and then cultured in SCPM containing 30% FCS for three days. For all treatment groups, the media was changed daily. *Low serum control *and *Activin A *cells were seeded in serum-free SCPM. Media was replaced with the following; serum-free SCPM on Day 1, SCPM containing 0.2% FCS on Day 2 and SCPM containing 2% FCS on Day 3; the media for *Activin A *cells on Days 1 to 3 was supplemented with 100 ng/ml Activin A. Click here for file

Additional file 2 **Cell proliferation index**. The number of cells counted when USSC grown in SCPM containing 0%, 1%, 2% or 30% FCS for seven days are expressed as a percentage of the number of cells seeded. <100% initial seeding density indicates cell death has occurred, while >100% initial seeding density indicates cell proliferation has occurred. Different letters denote statistical significance (*P *< 0.05). Click here for file

Additional file 3 **Activin receptor (ACVR) protein levels in USSC grown in serum free conditions**. Representative light micrographs depicting the localization of ACVR (brown staining) in USSC grown in serum free conditions (SCPM containing 0% FCS; 400×). Nuclei are counterstained blue with Haematoxylin. USSC 1 (left column) and USSC 2 (right column) expressed ACVR1b (**A, B**), ACVR2a (**C, D**) and ACVR2b (**E, F**). No staining was detected in the IgG controls (**G, H**). Click here for file
