# Supplementary material for: Stimulation of Activin A/Nodal signaling is insufficient to induce definitive endoderm formation of cord blood-derived unrestricted somatic stem cells
Source: Stem Cell Res Ther. 2011 Apr 4;2(2):16. doi: 10.1186/scrt57 (PMC3226287; doi:10.1186/scrt57)
Supplement: Additional file 1 — Activin A induction strategy. Cells were divided into one of three treatment groups; Normal serum control, Low serum control and Activin A. Normal serum control cells were seeded in SCPM containing 30% FCS and then cultured in SCPM containing 30% FCS for three days. For all treatment groups, the media was changed daily. Low serum control and Activin A cells were seeded in serum-free SCPM. Media was replaced with the following; serum-free SCPM on Day 1, SCPM containing 0.2% FCS on Day 2 and SCPM containing 2% FCS on Day 3; the media for Activin A cells on Days 1 to 3 was supplemented with 100 ng/ml Activin A. [file scrt57-S1.PDF]

	Normal serum control	Low serum control	Activin A
Seed cells	SCPM + 30% FCS	SCPM + 0% FCS	SCPM + 0% FCS
Day 1	SCPM + 30% FCS	SCPM + 0% FCS	SCPM + 0% FCS<br>100ng/ml Activin A
Day 2	SCPM + 30% FCS	SCPM + 0.2% FCS	SCPM + 0.2% FCS<br>100ng/ml Activin A
Day 3	SCPM + 30% FCS	SCPM + 2% FCS	SCPM + 2% FCS<br>100ng/ml Activin A
