# Supplementary figures and images for: Stimulation of Activin A/Nodal signaling is insufficient to induce definitive endoderm formation of cord blood-derived unrestricted somatic stem cells
Source: Stem Cell Res Ther. 2011 Apr 4;2(2):16. doi: 10.1186/scrt57 (PMC3226287; doi:10.1186/scrt57)

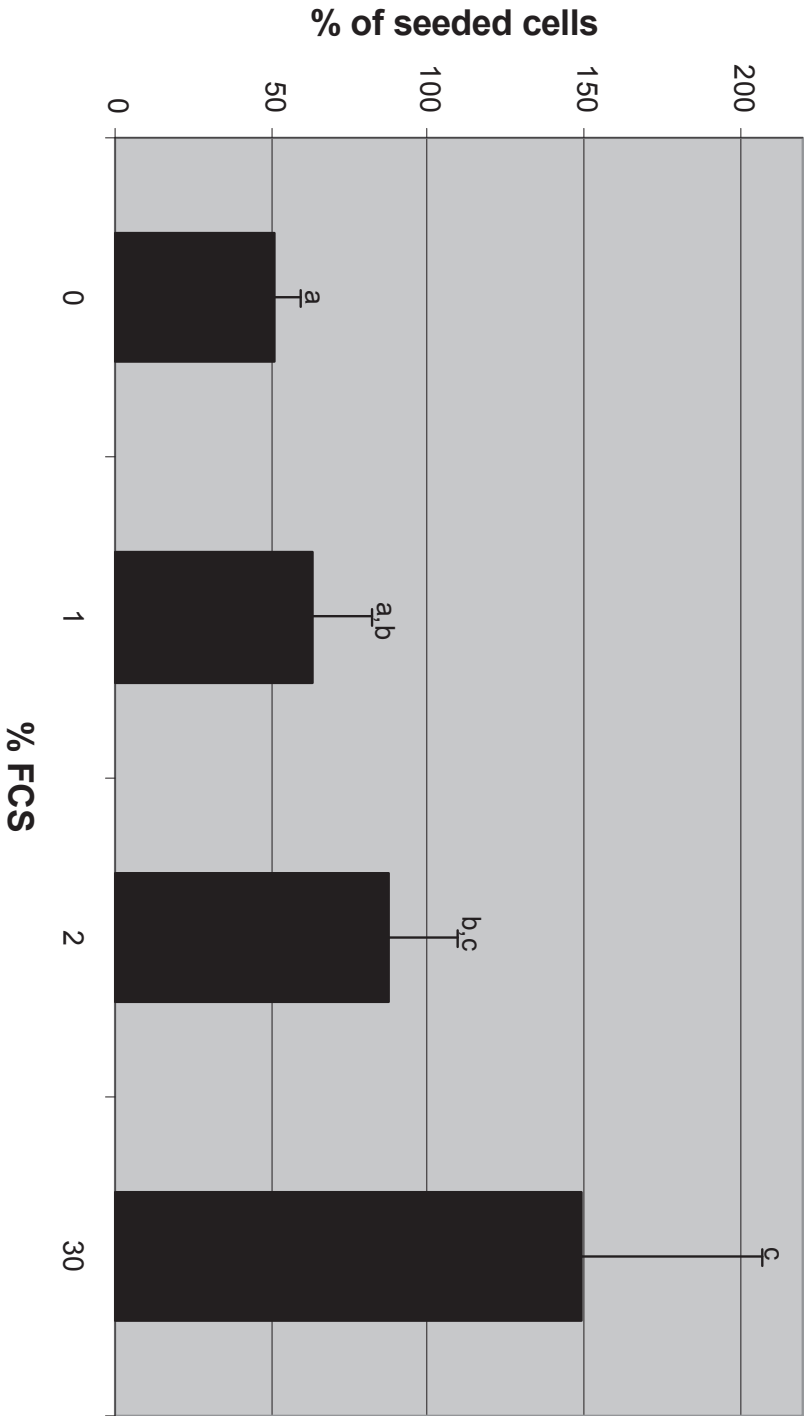

Supplement: Additional file 2 — Cell proliferation index. The number of cells counted when USSC grown in SCPM containing 0%, 1%, 2% or 30% FCS for seven days are expressed as a percentage of the number of cells seeded. <100% initial seeding density indicates cell death has occurred, while >100% initial seeding density indicates cell proliferation has occurred. Different letters denote statistical significance (P < 0.05). [file scrt57-S2.PDF]

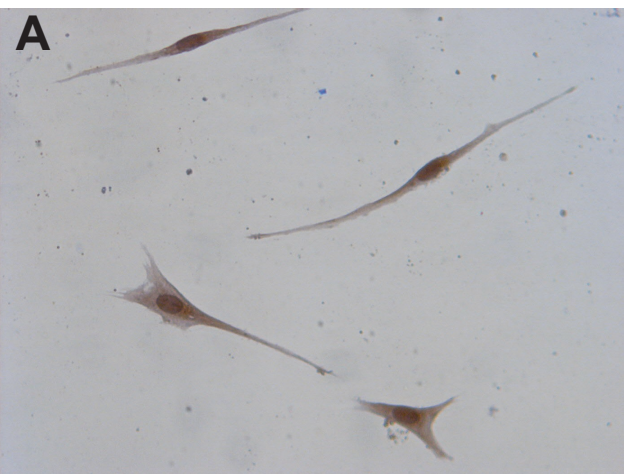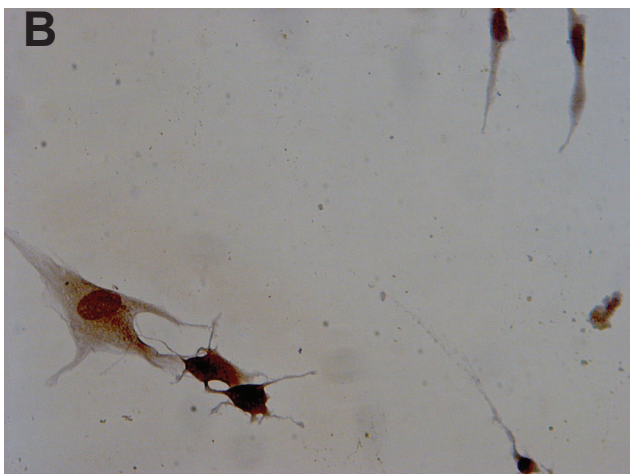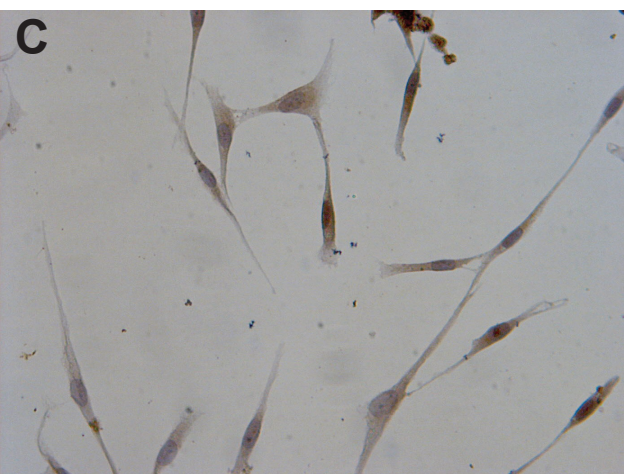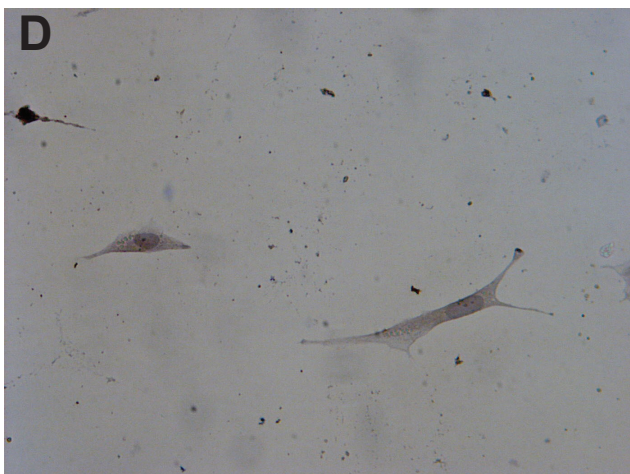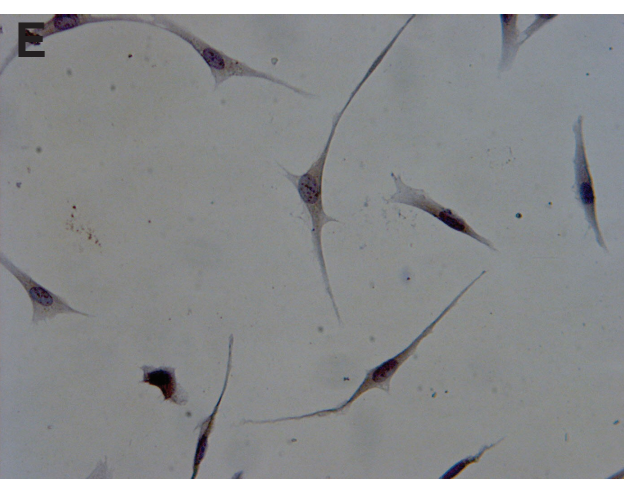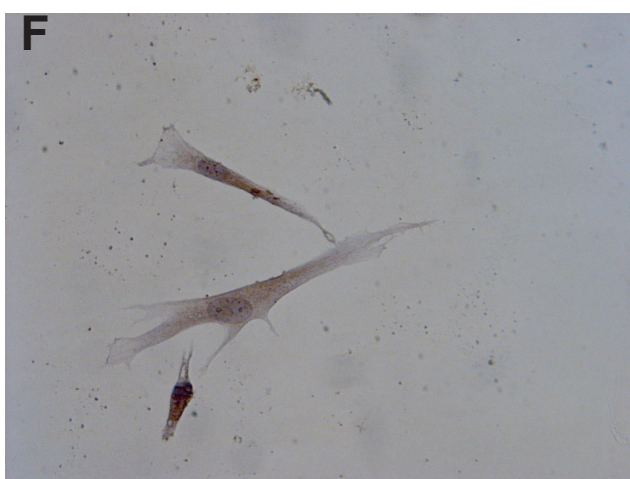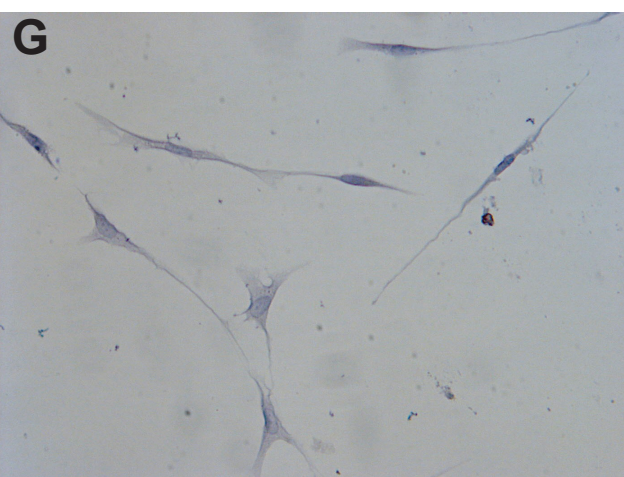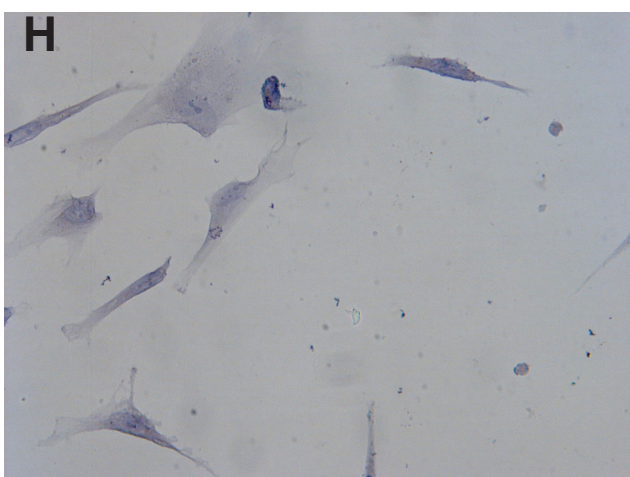

Supplement: Additional file 3 — Activin receptor (ACVR) protein levels in USSC grown in serum free conditions. Representative light micrographs depicting the localization of ACVR (brown staining) in USSC grown in serum free conditions (SCPM containing 0% FCS; 400×). Nuclei are counterstained blue with Haematoxylin. USSC 1 (left column) and USSC 2 (right column) expressed ACVR1b (A, B), ACVR2a (C, D) and ACVR2b (E, F). No staining was detected in the IgG controls (G, H). [file scrt57-S3.PDF]
